# Aorto-cavitary fistula: a rare case of *Enterobacter cloacae* infective endocarditis complicated by aortic root abscess and fistula to the right ventricle

**DOI:** 10.1093/jscr/rjac075

**Published:** 2022-03-22

**Authors:** Charis Tan, Kathryn M Wales, Annie Huynh, Linna Huang, Madeleine De Boer, Paul G Bannon, Matthew S Bayfield

**Affiliations:** Department of Cardiothoracic Surgery, Royal Prince Alfred Hospital, Sydney, Australia; Department of Surgery, University of New South Wales, Sydney, Australia; Department of Cardiothoracic Surgery, Royal Prince Alfred Hospital, Sydney, Australia; Department of Cardiothoracic Surgery, Royal Prince Alfred Hospital, Sydney, Australia; School of Medicine and Public Health, University of Newcastle, Newcastle, Australia; Department of Cardiothoracic Surgery, Royal Prince Alfred Hospital, Sydney, Australia; Department of Cardiothoracic Surgery, Royal Prince Alfred Hospital, Sydney, Australia; Sydney Medical School, Discipline of Surgery, University of Sydney, Sydney, Australia; Department of Cardiothoracic Surgery, Royal Prince Alfred Hospital, Sydney, Australia

## Abstract

*Enterobacter cloacae* are a rare cause of infective endocarditis (IE). We present an interesting case of a 51-year-old intravenous drug user with *E. cloacae* IE of a prosthetic aortic valve and a fistula into the right ventricle. He underwent surgical repair and 6 weeks of intravenous meropenem.

## INTRODUCTION

Demonstrating the evolving nature of infective endocarditis (IE), *Enterobacter cloacae* vegetation in a prosthetic valve and the resultant development of an aorto-cavitary fistula has proven to be a unique case.

## CASE REPORT

A 51-year-old male intravenous drug user (IVDU) presented to the Emergency Department with meningism and associated night sweats and nausea. Physical examination revealed only positive findings of a systolic murmur and left upper abdominal tenderness, with an unremarkable respiratory and neurological examination in the context of previous *Burkholderia cepacia* IE requiring a tissue aortic valve replacement (AVR) and antimicrobial sterilization, hepatitis C, provoked pulmonary embolism and alcohol withdrawal seizures.

**Figure 1:**
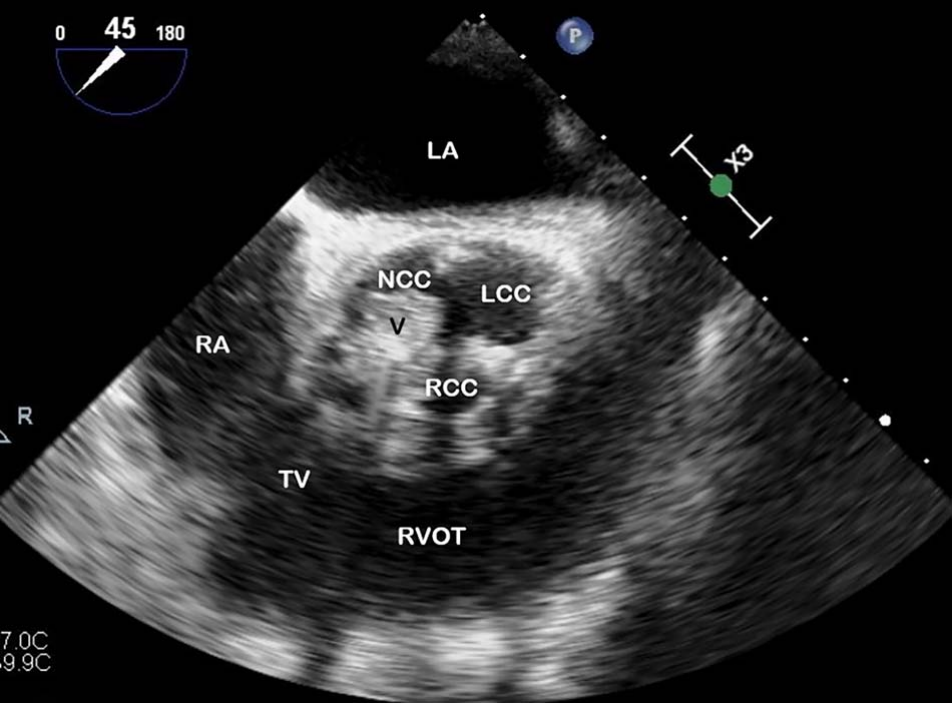
TOE, short axis demonstrating a vegetation (V) on the right coronary cusp (RCC; 11 × 8 mm) and on the non-coronary cusp (NCC; 15 × 11 mm); LCC, left coronary cusp; LA, left atrium; RA, right atrium; TV, tricuspid valve; RVOT, right ventricular outflow tract.

**Figure 2:**
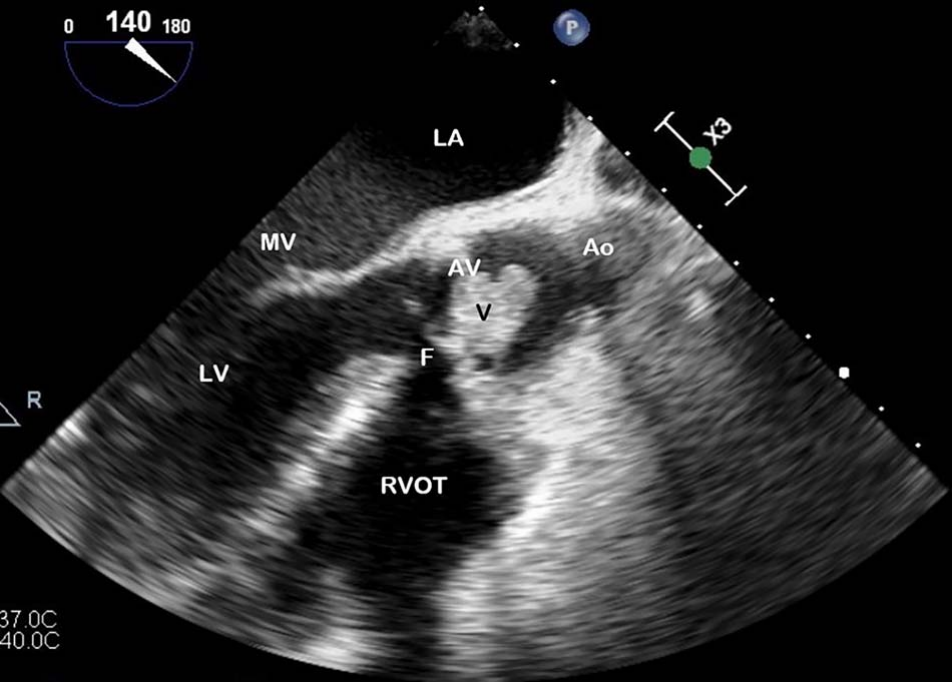
TOE, long axis demonstrating a vegetation (V) on the AV obstructing the left ventricular outflow tract; Ao, aorta; F, fistula; LV, left ventricle; MV, mitral valve.

The patient’s electrocardiogram demonstrated a prolonged PR interval, non-specific anterior ST changes and inferior T-wave inversion. Though the chest X-ray was unremarkable, concurrently elevated inflammatory markers urged empiric antibiotic commencement. Computed tomography imaging demonstrated hyperdensity in the subarachnoid space overlying the right cerebral hemisphere. The clinical suspicion of endocarditis prompted a transoesophageal echocardiogram (TOE), demonstrating multiple vegetations on the prosthetic aortic valve (AV) and features suggestive of a paravalvular abscess (Figs 1 and 2). Splenic and bilateral renal infarcts were confirmed. Multiple sets of blood cultures isolated *E. cloacae* and intravenous meropenem was commenced.

**Figure 3:**
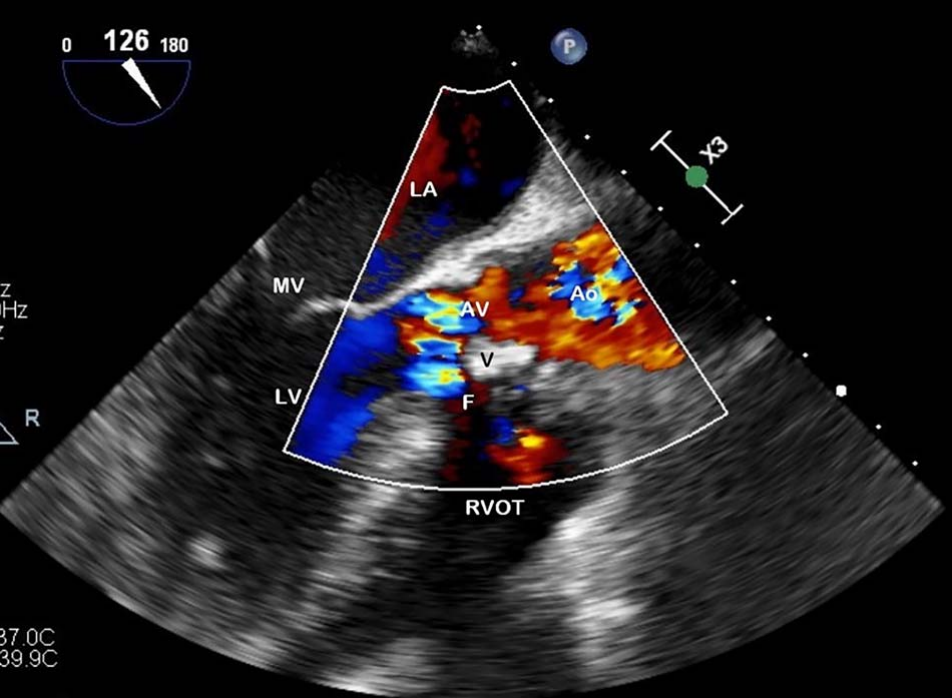
Preoperative TOE, long axis with colour flow highlighting the vegetation (V) obstructing flow through the AV and the fistula (F) which has developed from left ventricular outflow tract to RVOT.

The patient developed complete heart block, and subsequently, Torsades de Pointe, requiring an isoprenaline infusion, pacing via a temporary venous pacing system (and removed post-operatively) and urgent coiling of a large 4.8 × 2.5 cm right middle cerebral artery mycotic aneurysm 1 day prior to his redo-valve surgery. A repeat TOE demonstrated severe aortic regurgitation, prosthetic valve abscess, dehiscing and fistulizing from the aortic root to right ventricle (RV) on colour-flow doppler (Fig. 3). Proceeding to surgery, on inspection of the aortic root—findings confirmed a largely destroyed AV, multiple valvular vegetations and fistulization from the aortic root just below his right coronary cusp to his RV. A redo-sternotomy was performed, central cannulation was utilized—cross-clamp and bypass times were 89 and 141 minutes, respectively. Abscess debridement and an AVR (#23 Carpentier-Edwards Perimount magna-ease) were undertaken, with the fistula closed with the valve sutures, and a post-operative TOE confirmed no persisting fistula or peri or para-valvular leak around the new AV prosthesis, indicating significant improvement. He was referred for counselling with the Drug and Alcohol Service during his admission with extensive planned follow-up to maintain abstinence from IV drug use. The patient remained in sinus rhythm, completed 6 weeks of antimicrobial therapy and discharged home with no issues on follow-up.

## DISCUSSION

IE was first described in the 19th century [1]. Despite advances in diagnostic imaging, antimicrobial therapies and surgery, this condition remains associated with high patient morbidity and mortality. The constantly evolving landscape of IE has made it particularly complex to treat [2]. The common causative organisms of endocarditis are staphylococcal, streptococcal and enterococcal species [3]. *Enterobacter cloacae* is an aerobic gram-negative rod and a very rare cause for IE, deviating from the usual suspects of HACEK (*Haemophilus* spp., *Aggregatibacter actinomycetemcomitans*, *Cardiobacterium hominis*, *Eikenella corrodens* and *Kingella kingae*) organisms [4]. As of 2012, there had only been 26 published cases of *E. cloacae* IE [5].

Precipitating factors for IE are important considerations for diagnosis and management. Intravenous drug use is a recognized cause of IE, with an annual incidence of 1–5% in this population. A retrospective study of all hospitalizations from IE in the USA in 2015 identified 29% was associated with intravenous drug use [6]. Right-sided valves are also most frequently involved in both the general and IVDU population [7]. Prior prosthetic valve replacement further increases the risk of IE, identified in 0.1–2.3 cases per year [1].

The repetitious injection of impure particulate matter into circulation through intravenous drug use stimulates an inflammatory response whereby fibronectin is deposited on the cardiac valves [8]. Cumulative subclinical endothelial damage predisposes the tissue to pathogen seeding and vegetation formation without underlying cardiac disease [8, 9]. Embolization to solid organs, as in this case, are recognized complications. The local inflammatory response can damage nearby valvular and perivalvular structures, disrupting cardiac anatomy predisposing the patient to erosion of the valvular annulus, mycotic aneurysms, pseudoaneurysms, abscess and fistula formation [10]. Prosthetic valves dramatically increase the likelihood of periannular extension of infection ranging from 56 to 100% of reported cases, increasing the risk of morbidity and mortality despite surgical therapy [11].

Specifically, the formation of aorto-cavitary fistulae is a relatively rare complication first reported in 1924 as an incidental finding on an autopsy [12]. Anguera *et al*. reported a retrospective, multi-centre descriptive study of 4681 episodes of IE, with 76 (1.6%) complicated by aorto-cavitary fistulae—the largest series we identified. They were described in 0.4% of IVDU endocarditis, 1.8% of native valve endocarditis and 3.5% of prosthetic valve endocarditis. In this case series, all IE was associated with AV involvement, but there is no preponderance for any specific cavity [3]. The presence of aorto-cavitary fistulae is associated with higher rates of heart failure, ventricular septal defect and atrioventricular block than non-ruptured abscesses [11].

## CONCLUSION

An evolving entity, shaped by intravenous drug use and previous vascular instrumentation and valvular replacement, IE remains a condition with high morbidity and mortality despite advances in diagnostics, surgical technique and antimicrobials.

We have presented a unique case of *E. cloacae* IE in a prosthetic AV resulting in development of an aorto-cavitary fistula and associated complications. To our knowledge, this patient represents the first reported case of *E. cloacae* causing an aorto-cavitary fistula.

## CONFLICT OF INTEREST STATEMENT

The authors declare that there is no conflict of interest.

## FUNDING

The authors received no financial support for the publication of this article.
